# High-resolution contact networks of free-ranging domestic dogs *Canis familiaris* and implications for transmission of infection

**DOI:** 10.1371/journal.pntd.0007565

**Published:** 2019-07-15

**Authors:** Jared K. Wilson-Aggarwal, Laura Ozella, Michele Tizzoni, Ciro Cattuto, George J. F. Swan, Tchonfienet Moundai, Matthew J. Silk, James A. Zingeser, Robbie A. McDonald

**Affiliations:** 1 Environment and Sustainability Institute, University of Exeter, Cornwall, United Kingdom; 2 Data Science Laboratory, Institute for Scientific Interchange Foundation, Torino, Italy; 3 Guinea Worm Eradication Programme, Ministry of Public Health, N’Djamena, Republic of Chad; 4 The Carter Center, Atlanta, Georgia, United States of America; Universidad Nacional Mayor de San Marcos, PERU

## Abstract

Contact patterns strongly influence the dynamics of disease transmission in both human and non-human animal populations. Domestic dogs *Canis familiaris* are a social species and are a reservoir for several zoonotic infections, yet few studies have empirically determined contact patterns within dog populations. Using high-resolution proximity logging technology, we characterised the contact networks of free-ranging domestic dogs from two settlements (n = 108 dogs, covering >80% of the population in each settlement) in rural Chad. We used these data to simulate the transmission of an infection comparable to rabies and investigated the effects of including observed contact heterogeneities on epidemic outcomes. We found that dog contact networks displayed considerable heterogeneity, particularly in the duration of contacts and that the network had communities that were highly correlated with household membership. Simulations using observed contact networks had smaller epidemic sizes than those that assumed random mixing, demonstrating the unsuitability of homogenous mixing models in predicting epidemic outcomes. When contact heterogeneities were included in simulations, the network position of the individual initially infected had an important effect on epidemic outcomes. The risk of an epidemic occurring was best predicted by the initially infected individual’s ranked degree, while epidemic size was best predicted by the individual’s ranked eigenvector centrality. For dogs in one settlement, we found that ranked eigenvector centrality was correlated with range size. Our results demonstrate that observed heterogeneities in contacts are important for the prediction of epidemiological outcomes in free-ranging domestic dogs. We show that individuals presenting a higher risk for disease transmission can be identified by their network position and provide evidence that observable traits hold potential for informing targeted disease management strategies.

## Author summary

For communicable infections, variations in contact rates determine the flow of disease through populations. Therefore, describing contact patterns within populations could help to better predict and prevent disease outbreaks. Free-ranging domestic dogs are susceptible to a number of zoonotic infections yet few studies have investigated their contact patterns. We describe high-resolution contact data for free-ranging dogs in rural Chad and simulate the transmission of an infection comparable to rabies. We show that epidemic outcomes are determined by the seeded individual’s network position, which was also correlated with ranging behaviour. This demonstrates that between-individual variation in the risks of spreading infection may be linked with observable traits that can help inform targeted management strategies.

## Introduction

Heterogeneity in contact rates is influential in the epidemiology of both human and non-human animal diseases. In principle, variation in the contact rates among individuals affects their risk of acquiring and/or transmitting infections [1,2]. Relationships between host social behaviour and the distribution of infections have been demonstrated in several wild animal host-pathogen systems, from tuberculosis in badgers *Meles meles* [3] and meerkats *Suricata suricatta* [4] to nematodes in Japanese macaques *Macaca fuscata* [5]. One driver of these relationships is the variation in contacts between individuals, which can influence the flow of infection through populations [6,7]. Therefore, in order to successfully manage some diseases, it is important to understand the dynamics of host contacts that facilitate the transmission of infection [8].

The number of infectious disease emergence events in humans has been increasing over time, and the majority of these are zoonotic in origin [9]. This may, in part, be associated with the domestication of animals, as evidence suggests that the number of shared pathogens (between humans and non-human animals) increases with the time since a species was domesticated [10]. This is because domestication increases the exposure of people and animals to a greater range of pathogens, and increases the risk of humans acquiring zoonotic infections [11]. If domestic animals are free-ranging, they are also more likely to interact with wild animals, further facilitating the transmission of disease between people and wildlife [12].

Dogs *Canis familiaris* are among the earliest domesticated animals and they share 16% of their known pathogens with humans [10] and 47% with wild mammals [13]. Amongst these pathogens is rabies, a viral zoonosis that poses a significant public health risk, responsible for approximately 59,000 human deaths annually [14] and primarily transmitted to humans through the saliva of an infected dog when they are bitten [15,16]. Mathematical models can be applied to inform management efforts by predicting epidemics and, for rabies, these models are relatively well developed [17]. However, one of the challenges identified in controlling rabies is a lack of information on dog ecology [18] and variation in contact rates has been identified as being especially influential for epidemic outcomes in a number of modelling studies [19,20]. This is unsurprising given that dogs are social animals that exhibit pronounced between-individual variation in their behaviour [21].

Collecting high resolution data on the contact rates between individuals is a major challenge, particularly for free-ranging animals. This lack of empirical data has meant that stochastic models have relied on assumptions that contact rates are density dependent or have included a frequency dependent function in the form of spatial and/or social scaling parameters to generate variation in the probability of contacts [20,22]. Although these assumptions are biologically sound, they fail to capture social phenomena that could influence disease transmission, such as assortative mixing [23] and clustering [24]. Including observed contact data in stochastic models of communicable diseases could help better predict epidemics at a local scale and help identify novel management techniques [25].

To date, there has been only one study published that integrated observed contact rates of free-ranging dogs into a model for the transmission of rabies [26], in which the contact network of dogs was characterised over 3.5 days in an urban environment. They found that urban dogs formed communities that were defined by roads, which acted as a barrier to movement. When simulating outbreaks of rabies, the authors observed that major epidemics were avoided when 70% of the population were vaccinated and targeted management based on network measures increased the effectiveness of vaccination. However, it is unclear if this would also apply to rural dog populations, where the landscape and dog-human relationships are likely to be different to that in an urban environment [27], where unowned dogs are rare, roads are few and where hunting, subsistence farming and fishing are more prevalent.

In this study, we used automated proximity loggers to generate high-resolution contact networks of free-ranging dogs in an area of rural sub-Saharan Africa, where dogs are susceptible to a number of zoonotic infections. We use these data to model the transmission of an infection that is epidemiologically similar to rabies. We test the effect of including observed heterogeneities in contacts between free-ranging dogs on predictions for epidemic size. Using a network model we simulate epidemics through random networks, the observed network characterised as binomial (present/absent) interactions and the observed network when weighted by the duration of interactions. The observed binomial network introduces non-random structures while maintaining uniformity and the observed weighted network adds non-random and non-uniform mixing. In addition, we investigate the effect of seeding different individuals with the infection. If contact heterogeneity influences epidemics it may be possible to predict epidemic outcomes using the network position and/or associated traits of the seeded individual.

## Methods

### Ethics statement

This study was approved by the University of Exeter College of Life and Environmental Sciences (Penryn Campus) Ethics Committee (Reference 2016/1488).

### Data collection

Dogs were studied between June 24^th^ and July 12^th^ 2016 in two settlements, each comprising two neighbouring villages, located along the Chari River in the Guelendeng district of the Mayo-Kebbi East region of Chad. The settlement Kakale is located to the south-east of Guelendeng town and includes the villages Kakale-Mberi (10°53'0.79"N, 15°38'8.45"E) and Awine (10°48'6.34"N, 15°37'56.61"E). Kakale-Mberi is a linear settlement along a main (dirt) road that runs parallel to the Chari River. Awine is a dispersed settlement that is seasonally occupied by the people of Kakale-Mberi, who move there to cultivate crops. The settlement Magrao is comprised of the villages Magrao and Sawata (centred on 10°59'44.31"N, 15°29'29.27"E), located to the north of Guelendeng. Magrao is a dispersed village lying between the Chari River and the main road from Guelendeng to the capital, N’Djamena. Sawata is a smaller village that is surrounded by Magrao but is distinguished by different ethnicity and a higher prevalence of pastoralism.

All dogs had clear ownership and were associated with a specific household. They were all sexually intact. With the consent of owners, dogs were collared with standard nylon dog collars (Ancol Heritage). Puppies (less than 6 months of age) were not collared. Collars were fitted with two devices; (1) an i-GotU GT-600 GPS unit (Mobile Action Technology Inc., Taiwan) and (2) a wearable proximity sensor based on a design developed by OpenBeacon project (http://www.openbeacon.org/) and the SocioPatterns collaboration consortium (http://www.sociopatterns.org/). The GPS units were configured with a fix interval of 10 minutes and a sleep mode to extend battery life. The proximity sensors exchange one ultra-low power radio packet per second in a peer-to-peer fashion and, have been successfully deployed in several studies on humans [28,29]. The exchange of radio-packets is used as a proxy for the spatial proximity of individuals wearing the sensors [30,31]. Close proximity is measured by the attenuation, defined as the difference between the received and transmitted power. The attenuation threshold used in this deployment was selected to detect close-contact events (within 1–1.5 m), during which a communicable disease infection might be directly transmitted, either by airborne transmission or by direct physical contact. Additional data collected on the individual dogs included sex and body condition score (BCS; [32]). Due to low frequencies of some scores, we categorised them into poor (BCS ≤ 2) and moderate (BCS ≥ 3). Interviews using a standardised questionnaire were carried out at households to record the number of dogs owned and the dogs’ ages, as recalled by the owner. A single observer estimated BCS and another conducted all household interviews. Dogs aged 12 months or less were classified as juveniles, dogs aged between 13 and 24 months were classed as sub adults and dogs older than 24 months were regarded as adults [33]. Since all households known to have dogs in the settlement were visited, the dog population size (excluding puppies) was calculated for each settlement by summing the reported number of owned dogs from each household.

### Data processing

The proximity data were extracted from devices and cleaned by identifying corrupted sensors (where no data were available) or anomalous signals (such as continuous bursts of data). The GPS data were cleaned by removing erroneous fixes with speeds greater than 20 km/hr between locations. For both GPS and proximity data we discarded records collected on the first and last day of collar deployment in each village; providing time for the dogs to habituate to collars at the start and to account for the collection of the collars at the end of the field study.

Data analysis was conducted in R v3.3.3 [34] and Python v2.7. The R packages ‘sp’ v1.2–3 and ‘rgdal’ v1.2–5 were used to project the GPS data into the relevant coordinate reference system for Chad (EPSG:32634). The package ‘adehabitatHR’ v0.4.14 was used to calculate the dog’s total range (99% minimum convex polygons) and core range (60% kernel density estimate).

Networks were treated as undirected symmetric networks. Since dogs were not collared for the same number of days, the weights for the weighted networks were converted to the average number of seconds the dogs were in contact per day monitored. This was done by dividing the total duration in seconds over which a pair was in contact, by the shorter of the two periods in days for which the two dogs were collared. These weights were then log_10_ transformed. The global and local network metrics were calculated using the R package ‘igraph’ [35]. The network position of individuals was described using metrics most relevant to disease transmission [36], including: degree (the number of unique connections of an individual), strength (the summed strength of all connections for an individual), betweenness (the number of shortest paths between other individuals upon which the focal individual lies), and eigenvector centrality (a measure of second order contacts whereby a higher score is assigned to individuals if they associate with highly connected individuals or many moderately connected individuals). To compute the probability density distribution of contact durations and the complementary cumulative distribution function (CCDF) of edge weights, we used the Python package ‘Powerlaw’ v1.4.1. Community membership describes individuals that are closely associated/clustered together and these groups were identified using the edge betweenness and Greedy algorithms in the Python package ‘igraph’ v0.7.1.

### Epidemic simulations

The package ‘Epimodel’ v1.3.0 [37] was used to build a Susceptible, Exposed, Infected and Removed (SEIR) network model of infection spread. Simulations were run on the observed binomial network, the observed weighted network and the null model (random networks). Random networks are traditionally used in network analysis to overcome the non-independence nature of contacts, and are typically constrained to biologically plausible scenarios. The null model for this study was that individuals mix randomly and so random networks were generated using the Erdős-Rényi model, conserving the observed number of nodes (individual dogs) and edges (connections). Every individual in the binomial and weighted networks was seeded with the infection and, for each seeded individual, 100 simulations of the model were run. For the null model, the same procedure was conducted, however, each simulation involved a different random network and all seeded individuals experienced the same set of 100 random networks. Simulations were run over 300 time steps (days). The network model assumed that (a) there was no recruitment or loss of individuals to the population (except the eventual removal of those infected), (b) the edges and weights of the network did not rewire over time or in response to infected or removed individuals and (c) individuals do not change their behaviour when infected.

For each simulation an initial seed (infectious individual) was selected at time step 1. At time steps 2–300, an edge list of infectious and susceptible individuals was made and transmission events were determined through a random binomial draw using the calculated per link transmission probability (β):

$$\beta =1-{\left(1-\lambda \right)}^{\alpha }$$ (1)

The probability of infection after being bitten (λ) was taken to be 0.49 [38]. To our knowledge, no data are available on the act rate (α; number of bites per partnership per day) of rabid dogs and it was therefore taken to be:

$$\alpha =\frac{\text{log}\left(1-\beta \right)}{\text{log}\left(1-\lambda \right)}$$ (2)

Where β was calculated by assuming a constant value of the basic reproductive number (R_0_) and by rearranging its definition in the heterogeneous mean-field approximation [39]:

$$\beta =\frac{R_{0}\mu \langle k\rangle }{\langle k^{2}\rangle -\langle k\rangle }$$ (3)

The mean degree 〈k〉 and mean square degree 〈k^2^〉 were extracted from the observed networks (see Table 1). The infectious period (μ) was randomly drawn from a gamma distribution (shape = 3.0; scale = 0.9; see [38 & 40]). Simulations were run for a range of basic reproductive numbers found in the literature for rabies in dogs. The lower R_0_ was set to 1.2, the mid value was 1.8 [38] and the upper R_0_ was 2.4 [41]. The transmission probability for different edge weights (β_*ij*_) was calculated using Eq 4:

$$\beta_{ij}=1-{\left(1-\lambda \right)}^{\alpha_{ij}}$$ (4)

$$\alpha_{ij}=\alpha \frac{w_{ij}}{1+w_{ij}}\times 2$$ (5)

**Table 1:** Summary of individual attributes and the global and local network metrics for free-ranging dogs from two settlements, Kakale and Magrao, in rural Chad.

	Kakale	Magrao
*Attributes*		
Sex (male: female)	25: 23	39: 21
Age (adult: sub adult: juvenile)*	25: 13: 9	26: 18: 15
BCS (poor: moderate)**	24: 24	15: 40
Core range (km^2^)***	2.28 ± 0.72	0.17 ± 0.05
Total range (km^2^)***	20.77 ± 3.27	4.56 ± 0.66
*Global network metrics*		
Nodes	48	60
Edges	160	191
Edge Density	0.14	0.11
Diameter	7	8
Average path length	3.01	3.57
Clustering coefficient	0.51	0.50
*Local network metrics*		
Degree	6.7 ± 0.5	6.4 ± 0.4
Square degree	55.4 ± 7.2	49.9 ± 6.3
Strength	10.2 ± 0.7	9.1 ± 0.7
Eigenvector	0.32 ± 0.05	0.19 ± 0.03
Betweenness	47.1 ± 9.9	75.9 ± 16.8

The mean ± standard error is reported for spatial attributes and the local network metrics. Total range is the 99% Minimum Convex Polygon and core range is the 60% Kernel Density Estimate. Global network metrics include the number of nodes (individuals), number of edges (connections between individuals), diameter (longest path length), average path length and cluster coefficient (transitivity). Local network metrics include the degree (number of connections), square degree, strength (summed strength of connections), Eigenvector centrality (second order contacts) and betweenness (contribution to number of shortest paths).

*The age of one individual in both settlements was unknown.

** In Magrao, data for BCS were missing for 5 individuals.

*** The spatial ranges of 9 individuals in Magrao and 3 individuals in Kakale were unknown.

The weighted act rate (α_ij_) was calculated through Eq 5 which is modified from Reynolds et al [7]. Here we assumed that α_ij_ was positively associated with the daily average of the total duration that individuals were in contact (*w*_*ij*_), and in so doing, we applied a sigmoidal scaling function. This value was then multiplied by two to shift the mean of β_ij_ to β. The use of this scaling function is justified where biting is the main method of rabies transmission and only a short contact time is required. Once a transmission event occurred, a random draw from a gamma distribution was used to allocate an incubation period (shape = 1.1; scale = 20.1; see [38 & 40]) and infectious period (see above for parameters). During the incubation period individuals were considered to be in the exposed category. Once the incubation period was over, the individual was classed as infected and could transmit the disease until such time as the assigned infectious period was over and the individual, along with its associated edges, was removed from the network. For this study, an epidemic was defined as disease transmission to at least one other individual.

### Statistical analysis

Differences in ranked network position (degree, strength, eigenvector centrality and betweenness) between nodal attributes (sex, age, BCS and home ranges) were identified by calculating t-statistics, using either t-tests or linear models. Observed statistics were compared to the distribution of test statistics from null models to identify if they were significantly different to those expected had individuals mixed randomly [42]. Null models consisted of 10,000 random networks generated by randomly shuffling the node attributes while keeping the structure of the observed network the same. Homophily within the attributes age, sex and household was investigated by calculating the assortativity (r) coefficient using the ‘assortnet’ package in R. Again the observed coefficients were compared to the distribution of coefficients from null models. To see if community membership was determined by the dogs’ sex, age or household, we used the Normalized Mutual Information (NMI) score to scale the results between 0 (no mutual information) and 1 (perfect correlation). To investigate if there was a correlation between edge existence/weight and the distance between households, the ‘sna’ package v.2.4 in R was used to perform a quadratic assignment procedure (QAP) with 1000 permutations.

Generalised additive models (GAMs) were used to identify non-linear relationships between the averaged epidemic outcomes of simulations for seeded individuals and their ranked network position (degree, eigenvector centrality, and betweenness). Models were fit with family set to Gaussian and included a smoothing term (k = 3). Strength was not investigated in these models since no difference in epidemic size between weighted and binomial simulations was observed. Since measures of network position are often correlated, separate models were fitted for each measure of centrality and type of network. Akaike’s Information Criterion (AIC) and adjusted r^2^ values were extracted and used to identify which centrality measure best explained epidemic outcomes.

## Results

### Network structure

**Fig 1:**
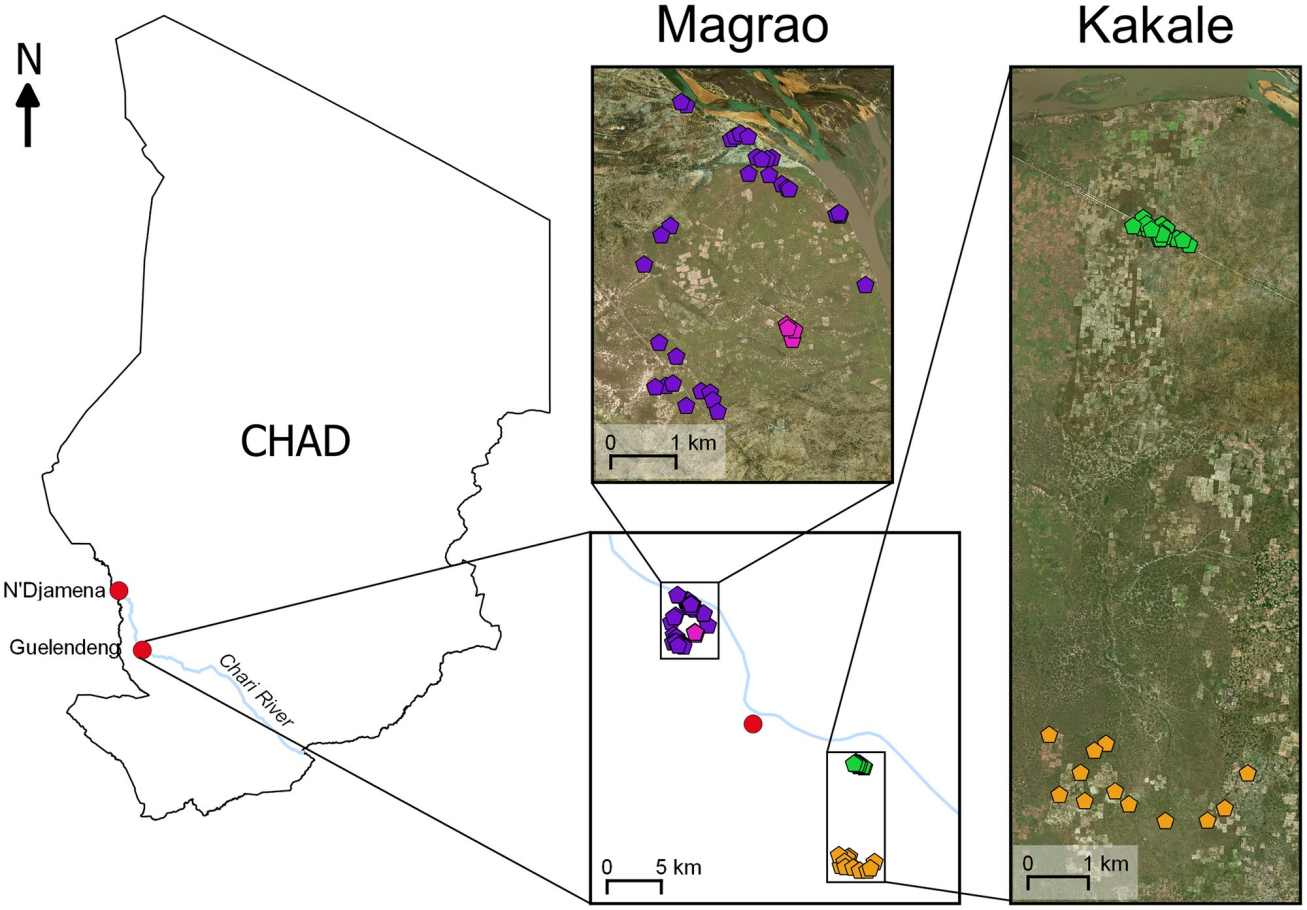
Locations of two settlements in rural Chad at which contact patterns of free-ranging domestic dogs were quantified. Pentagons represent a household where at least one dog was collared. Villages include Magrao (purple), Sawata (pink), Kakale-Mberi (green) and Awine (orange). The satellite image was generated using the Esri world imagery basemap (sources: Esri, DigitalGlobe, GeoEye, i-cubed, USDA FSA, USGS, AEX, Getmapping, Aerogrid, IGN, IGP, swisstopo, and the GIS User Community).

In Kakale, collars were successfully deployed for a mean of 8 days (range 2–9 days) on 48 dogs (86% of the population excluding puppies) from 28 different households (Fig 1). The distance between dog owning households ranged from 23–10,002 m. 8561 contact events were recorded between dogs in Kakale and the median contact duration was 20 seconds with a percentile (2.5% - 97.5%) range of 20–200 seconds. In Magrao, contact data were collected for a mean of 8 days (7–10 days) for 60 dogs (82% of the population) from 36 households. The distance between households ranged from 35–4758 m. 7361 contact events were recorded between dogs in Magrao and the median contact duration was 20 seconds, with a percentile range of 20–160 seconds.

**Fig 2:**
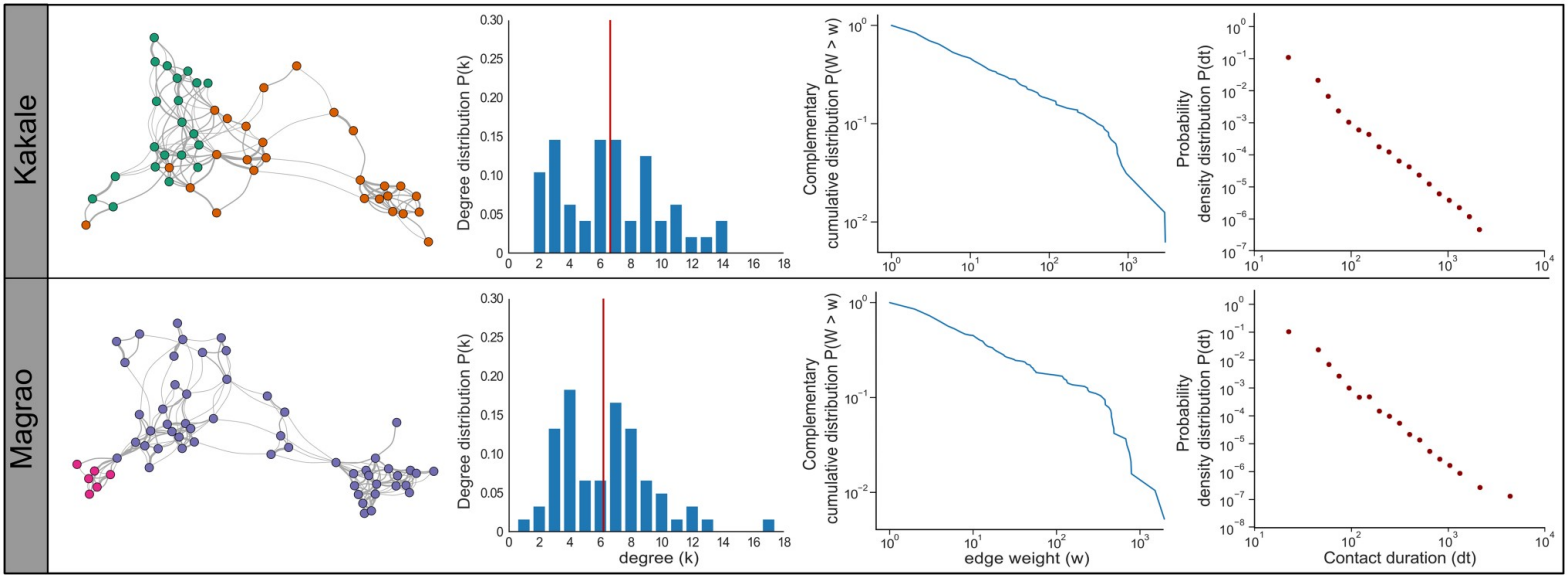
The contact networks, degree distribution, edge weight distribution and probability density distribution of contacts between free-ranging dogs for two settlements in Chad. In the networks, the circles represent individuals and the colours indicate the village that the dogs belong to: Kakale-Mberi in green, Awine in orange, Magrao in purple and Sawata in pink. The lines connecting individuals indicate that they have been in contact and the thickness of the lines are proportional to the logged daily average contact time between individuals. The red line of the degree distributions (probability that a randomly chosen node has degree ≥ k) indicates the mean degree (number of connections).

The global structure of both networks revealed high levels of clustering and short average path lengths (Table 1). Furthermore, community analysis using the edge betweenness (EB) and Greedy (G) algorithms showed the dog populations in both settlements exhibited high modularity in the binomial network (Kakale: EB = 0.48, G = 0.51; Magrao: EB = 0.56, G = 0.57) and the weighted network (Kakale: EB = 0.57, G = 0.603; Magrao: EB = 0.60, G = 0.617). Magrao was the larger of the two networks and had a wider degree distribution (k_min_ = 1, k_max_ = 17) than that of Kakale (k_min_ = 2, k_max_ = 14). In both networks the degree distribution was homogenous (Kakale: coefficient of variation (CV) = 0.49, Magrao: CV = 0.48) while the distributions for the duration of contacts were highly heterogeneous (Kakale: CV = 1.88, Magrao: CV = 1.85), and the probability density distribution declined as contact durations increased (Fig 2).

### Individual attributes and network position

Dogs in Magrao had substantially smaller ranges than dogs from Kakale, and the distribution of ranges was right skewed for both settlements (S1 Fig). Dogs in Kakale that had larger ranges had higher ranked eigenvector centralities and this was significantly different to null models (Table 2). Similarly, the home ranges of dogs in Kakale were positively correlated with their ranked degree, and this correlation was significantly greater than that of null models. In both networks, comparisons to null models revealed no significant association of any ranked network measures (degree, strength, eigenvector centrality or betweenness) with sex, age or body condition.

**Table 2:** Relationships between the ranked network position of free-ranging domestic dogs from two rural settlements in Chad and their individual attributes.

	Ranked Degree		Ranked Strength		Ranked Eigenvector Centrality		Ranked Betweenness	
Settlement	t	p	t	p	t	p	t	p
	*Male vs Female*							
Kakale	-0.755	0.222	-0.237	0.404	-0.113	0.446	1.796	0.043
Magrao	1.735	0.045	1.530	0.069	1.060	0.147	-0.118	0.456
	*Adult vs Juvenile*							
Kakale	2.073	0.031	1.580	0.072	1.148	0.131	2.039	0.033
Magrao	0.754	0.233	-0.578	0.276	1.270	0.109	1.632	0.054
	*Adult vs Sub Adult*							
Kakale	0.501	0.306	1.258	0.106	0.048	0.484	0.012	0.488
Magrao	-0.905	0.490	-0.620	0.303	-0.013	0.494	1.002	0.161
	*Sub Adult vs Juvenile*							
Kakale	1.238	0.115	0.350	0.367	0.934	0.425	1.590	0.065
Magrao	0.480	0.237	0.905	0.452	1.363	0.124	0.456	0.316
	*BCS (Moderate vs Poor)*							
Kakale	1.266	0.103	1.570	0.057	0.061	0.472	0.041	0.481
Magrao	0.660	0.259	-0.205	0.414	1.921	0.033	0.760	0.224
	*Core Range*							
Kakale	3.603	**<0.001**	2.044	0.024	3.895	**<0.001**	-1.372	0.007
Magrao	0.822	0.217	0.232	0.420	0.508	0.317	1.897	0.029
	*Total Range*							
Kakale	2.936	**0.003**	1.708	0.048	3.915	**< 0.001**	2.310	0.012
Magrao	1.035	0.164	0.116	0.459	0.703	0.243	2.403	0.008

Observed statistics for differences in ranked degree (number of connections), strength (summed strength of connections), Eigenvector centrality (second order contacts) and betweenness (contribution to number of shortest paths) are reported. Total ranges are based on 99% Minimum Convex Polygons and core ranges are 60% Kernel Density Estimates. P-values are for comparisons between the t-statistics of the observed and random graphs. P-values in bold are significant. The alpha level was corrected for multiple comparisons using the Bonferroni correction (α = 0.007).

All measures of community membership were strongly correlated with household membership in both the binomial networks (Kakale: NMI_EB_ = 0.622, NMI_G_ = 0.625; Magrao: NMI_EB_ = 0.739, NMI_G_ = 0.649) and weighted networks (Kakale: NMI_EB_ = 0.674, NMI_G_ = 0.70; Magrao: NMI_EB_ = 0.725, NMI_G_ = 0.713). Community membership had no significant relationship with either the dog’s sex or age (S1 Table). When compared to null models, dogs in both settlements had a strong preference to associate with individuals from the same household and no assortative mixing patterns were found between dogs of a different/similar age or sex (Table 3). QAP tests found a significant negative correlation for the distance between households and the existence of an edge (Kakale: r = -0.23, p < 0.001; Magrao: r = -0.4, p < 0.001). A negative correlation was also found for the relationship between household distance and edge weight (S2 Fig Kakale: r = -0.22, p < 0.001; Magrao: r = -0.37, p < 0.001).

**Table 3:** The binomial and weighted assortativity for the contact networks of free-ranging dogs from two settlements, Kakale and Magrao, in rural Chad.

Attribute	Settlement	Binomial		Weighted	
		r	p	r	p
Sex	*Kakale*	-0.051	0.381	-0.091	0.237
	*Magrao*	-0.075	0.206	-0.160	0.027
Age	*Kakale*	0.060	0.112	0.047	0.205
	*Magrao*	-0.015	0.405	-0.013	0.447
Household	*Kakale*	0.130	**< 0.001**	0.283	**< 0.001**
	*Magrao*	0.162	**< 0.001**	0.329	**< 0.001**

The assortativity between individuals of a similar sex, age and household in Kakale and Magrao. The r coefficient is for the observed network and the p-values are for the comparison between the observed coefficient and the distribution of those from null models. Significant p-values are in bold.

### Epidemic simulations

**Fig 3:**
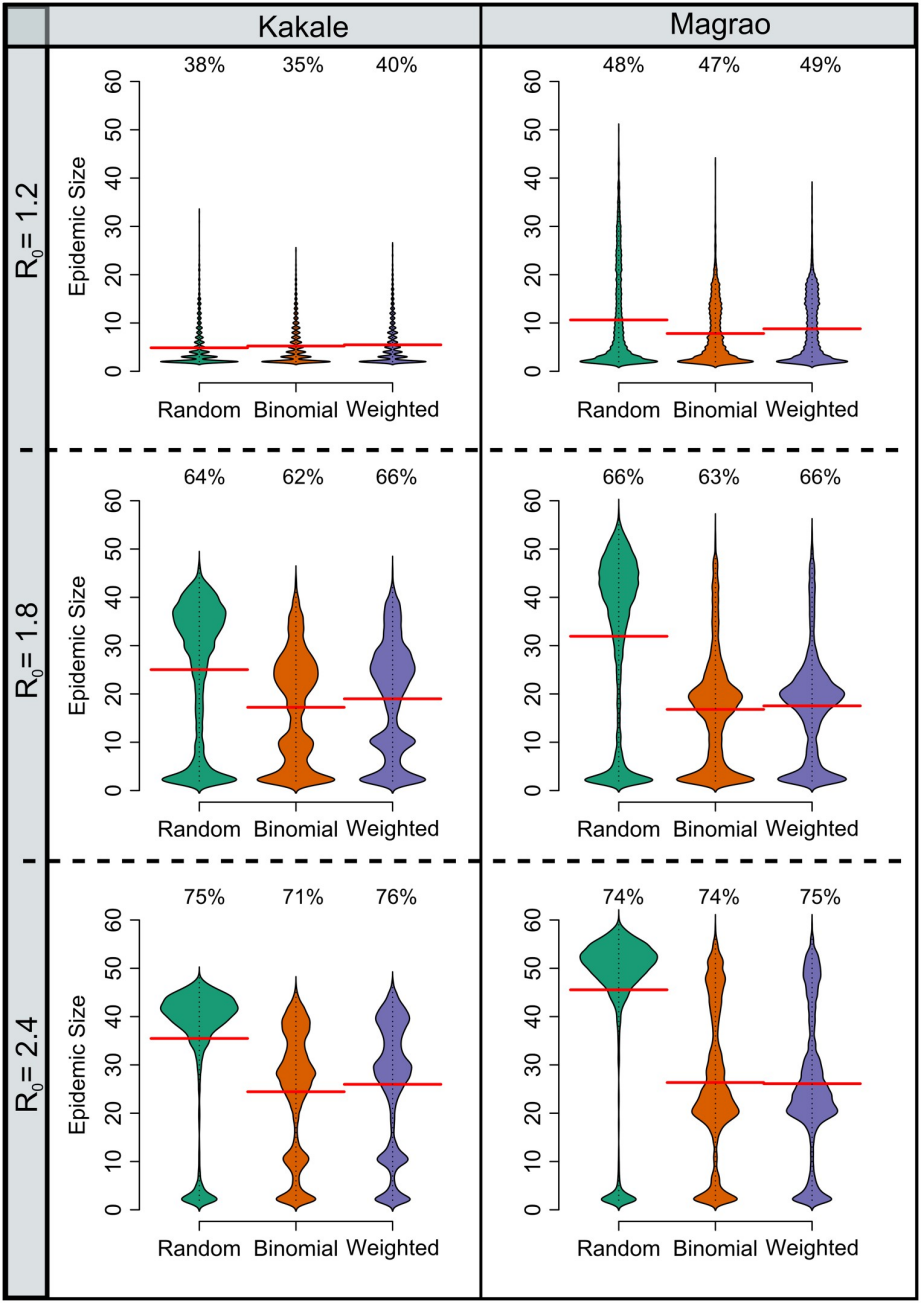
Simulated epidemic sizes of disease transmission through empirically determined contact networks for free-ranging dogs in two rural settlements in Chad. Bean plots show the distribution of epidemic sizes of simulations using the observed binomial and weighted networks and random networks: Kakale (n = 4800) and Magrao (n = 6000). All plots consider simulations where an epidemic occurred (the disease spread to at least one individual). The percentage of simulations that resulted in an epidemic is displayed above each bean plot. The horizontal red lines indicate mean epidemic size.

For both settlements, larger R_0_ values resulted in an increased risk of epidemics occurring and larger epidemic sizes (Fig 3, see S3 Fig for the frequency distributions of secondary cases). In simulations when R_0_ was 1.8 or 2.4, mean epidemic size was higher for random networks than that of simulations with observed contacts. Epidemic sizes for simulations using these random networks had a bimodal distribution, whereby epidemics either involved a large number of individuals or very few. In contrast, the distribution of epidemic sizes for observed networks had multiple peaks at intermediate sizes. The distributions of epidemic sizes differed for the two settlements, whereby Kakale had more intermediate peaks. Simulations with the lowest R_0_ value (1.2) showed no discernible difference in mean epidemic sizes between the random and observed networks.

**Fig 4:**
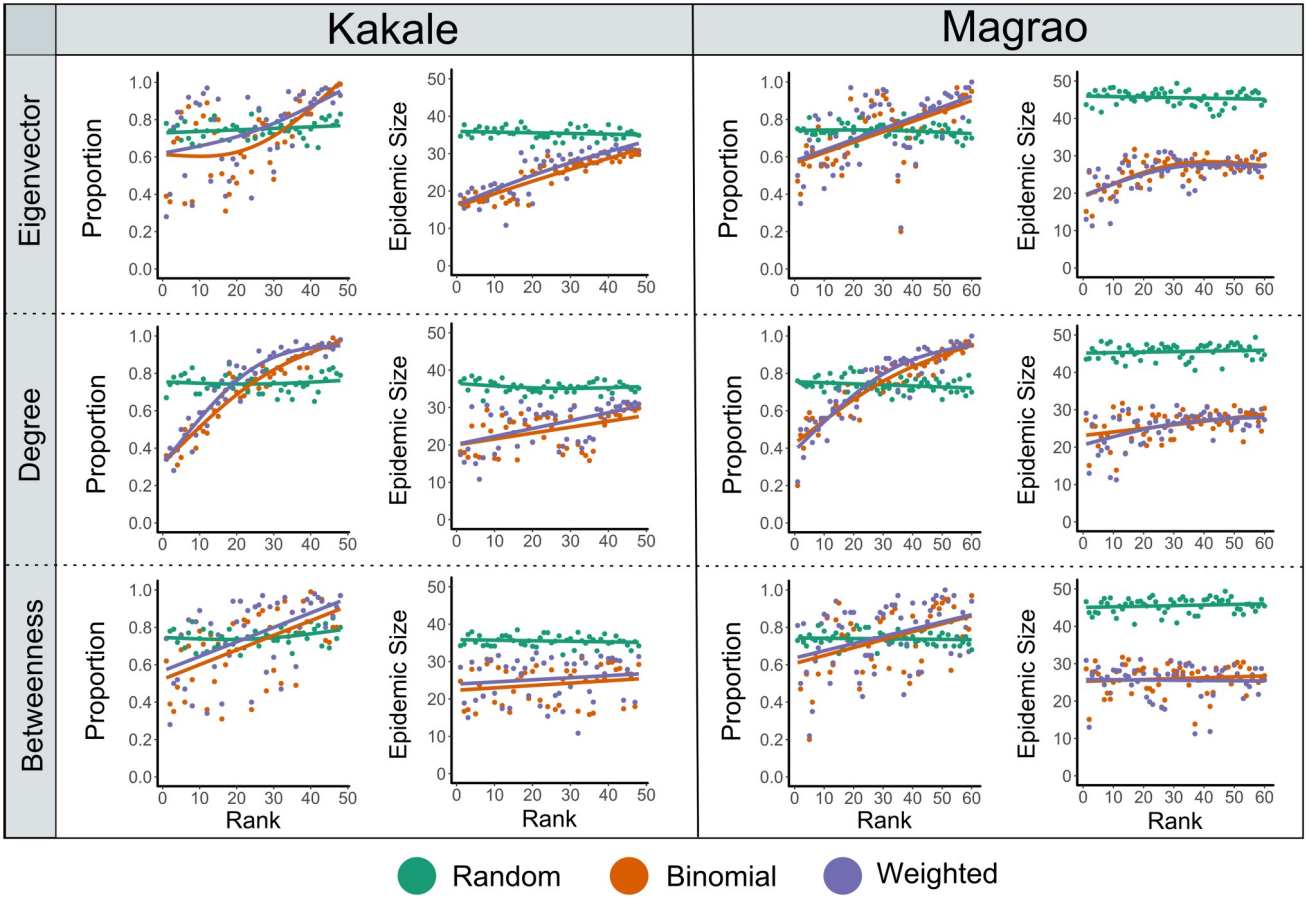
The relationship between epidemic outcomes simulated on contact networks of free-ranging dogs from two rural settlements in Chad and the seeded individual’s ranked network position. Scatter plots for each settlement (Kakale and Magrao) show the seeded individual’s ranked centrality measures (Eigenvector centrality (second order contacts), degree (total number of contacts) and betweenness (contribution to number of shortest paths)) plotted against the proportion of simulations that resulted in an epidemic (the disease was transmitted to at least one individual) and mean epidemic size. The mean epidemic sizes exclude simulations where the infection did not spread beyond the seeded individual. The data include the results for the random, binomial and weighted networks, and are for simulations when R_0_ was set to 2.4. GAMs are fitted to the data to identify non-linear trends.

For the observed networks for both settlements, the seeded individual’s ranked centrality measures (degree, eigenvector centrality and betweenness) were all positively correlated with the proportion of simulations that resulted in an epidemic (S4 Fig). The seeded individual’s ranked degree was the best predictor for the proportion of simulations to result in an epidemic (Table 4), and at larger R_0_ values the relationship between ranked degree and an epidemic outcome began to plateau for higher ranked individuals (Fig 4). As expected, the seeded individual’s observed centrality measures did not correlate with the proportion of simulations to result in an epidemic in any of the random networks.

**Table 4:** Measures of model fit for the relationship between epidemic outcomes simulated on contact networks of free-ranging dogs and the seeded individual’s network position. Networks were described in two settlements, Kakale and Magrao, in rural Chad.

	R_0_	Network	Proportion of simulations that resulted in an epidemic						Mean epidemic size					
			Eigenvector centrality		Degree		Betweenness		Eigenvector centrality		Degree		Betweenness	
			r^2^	AIC	r^2^	AIC	r^2^	AIC	r^2^	AIC	r^2^	AIC	r^2^	AIC
Kakale	1.2	Random	0	-130	0.03	-131	0.07	-133	0.13	99	0.02	105	0	106
		Binomial	0.45	-72	**0.93**	**-169**	0.33	-64	**0.49**	**120**	0.18	144	0.04	151
		Weighted	0.32	-53	**0.86**	**-130**	0.42	-61	**0.56**	**132**	0.31	153	0.03	170
	1.8	Random	0	-166	0	-166	0	-166	0.01	206	0	207	0	207
		Binomial	0.36	-35	**0.92**	**-138**	0.36	-36	**0.68**	**234**	0.09	284	0	288
		Weighted	0.26	-31	**0.91**	**-132**	0.34	-37	**0.71**	**231**	0.24	277	0	291
	2.4	Random	0.03	-151	0	-149	0.09	-153	0.01	178	0.05	176	0	178
		Binomial	0.40	-40	**0.94**	**-148**	0.29	-32	**0.79**	**219**	0.18	285	0.01	294
		Weighted	0.24	-27	**0.91**	**-132**	0.28	-30	**0.69**	**253**	0.25	295	0	308
Magrao	1.2	Random	0	-197	0.06	-201	0	-197	0	233	0	232	0.15	223
		Binomial	0.33	-59	**0.87**	**-158**	0.14	-45	**0.73**	**188**	0.34	243	0	268
		Weighted	0.37	-58	**0.88**	**-159**	0.16	-40	**0.68**	**223**	0.36	264	0	292
	1.8	Random	0	-199	0	-199	0	-199	0	259	0.03	257	0	259
		Binomial	0.35	-53	**0.91**	**-173**	0.18	-39	**0.66**	**262**	0.30	304	0	325
		Weighted	0.29	-47	**0.85**	**-138**	0.10	-33	**0.54**	**292**	0.33	314	0	338
	2.4	Random	0.02	-222	0.05	-225	0	-221	0	255	0	255	0	255
		Binomial	0.34	-68	**0.87**	**-164**	0.19	-55	**0.49**	**299**	0.17	327	0	339
		Weighted	0.30	-53	**0.87**	**-151**	0.13	-40	**0.30**	**333**	0.24	338	0	355

The adjusted r^2^ and AIC of fitted GAMs are reported for the seeded individuals centrality measures (Eigenvector centrality (second order contacts), degree (total number of connections) and betweenness (contribution to the number of shortest paths)). Results are reported for when R_0_ was set to 1.2, 1.8 and 2.4, and for the random, binomial and weighted networks of both Kakale and Magrao. The best r^2^ and AIC are highlighted in bold for each R_0_ of the binomial and weighted networks.

The seeded individual’s ranked eigenvector centrality and ranked degree were positively correlated with the mean epidemic size in simulations on the binomial and weighted networks for both settlements (S5 Fig). Ranked eigenvector centrality was the best predictor of mean epidemic size (Table 4), and for simulations of Magrao at larger R_0_ values, mean epidemic size plateaued for individuals with a higher ranked eigenvector centrality (Fig 4). The distributions of eigenvector centralities for dogs in each settlement (S6 Fig), were similar to the distribution of epidemic sizes in respective settlements. No correlation between the seeded individual’s network position and mean epidemic size was found in any of the random networks.

## Discussion

We have gathered high-resolution data on the contacts among free-ranging domestic dogs living in two rural settlements in Chad, an area where rabies infection is endemic and regularly causes human fatalities. Using these data we have demonstrated the importance of including observed contact patterns when simulating the transmission of an infection comparable to rabies. We show that the observed contact rates between dogs are heterogeneous and that interactions were dominated by contacts that were short in duration and between dogs from the same household. In our model, for the transmission of infection, the inclusion of observed contact rates resulted in fewer epidemics occurring compared to when random mixing was assumed and, for all but the lowest R_0_ values, epidemics were smaller in simulations using the observed networks. We also show that the seeded individual’s first and second order contacts were strong indicators of epidemic outcomes, verifying that individuals differ in the risk they present for the transmission of infections. Furthermore, for dogs in one settlement, second order contacts were correlated with ranging behaviour, suggesting that observable traits exist which could inform targeted management strategies.

The transmission probabilities associated with the lowest R_0_ value rarely resulted in an epidemic and, when one occurred, no more than a few individuals were infected. This meant that there was little difference in the overall mean epidemic size between simulations of random and observed networks. However, heterogeneity in contacts was still important in determining epidemic outcomes whereby the seeded individual’s ranked degree was positively correlated with the proportion of simulations that resulted in an epidemic, and this was echoed in simulations with higher R_0_ values. This finding demonstrates that, regardless of the transmission probability, dogs that are in contact with more individuals relative to the rest of the population are at higher risk of causing an epidemic should they become infected.

In simulations with all but the lowest R_0_ value, the risk of a large epidemic was higher when infection started in dogs with a higher ranked eigenvector centrality, and this was further emphasised where the distribution of eigenvector centralities paralleled that of epidemic sizes for each settlement. The importance of an individual’s eigenvector centrality in disease dynamics has also been shown in models for the transmission of *Mycobacterium bovis* in badgers [43] and observed parasite infection in Japanese macaques [5], where this measure was positively correlated with infection status. It appears that eigenvector centrality is a robust predictor of epidemic size and infection status because it describes how an individual is rooted into the network beyond their immediate connections.

We show that ranging behaviour was correlated with eigenvector centrality, but this was only true for dogs in Kakale. Both range sizes and eigenvector centralities were higher for dogs in Kakale than those in Magrao. This is likely due to anthropogenic variation in dog behaviour whereby during the study some people in Kakale moved with their dogs back and forth between a permanent residence and a seasonally-occupied homestead, while people in Magrao tended to stay at one. The dogs that accompany their owners in travelling between permanent and seasonal homesteads will have larger ranges and this would influence the dog’s network position by creating new contact opportunities. Nevertheless, the relationships between dog network position and epidemic outcomes were the same in both settlements. We also show that the distribution of dog owning households is important in determining contacts between dogs, with dogs more likely to have been in contact with and having stronger connections with dogs from closer households. However, it is important to note that this distance effect cannot fully explain the structure of the contact networks as many dogs from households in close proximity did not come into contact (S2 Fig). Although the dogs in this study were free-ranging, they were owned and anthropogenic influences on dog contact rates and ranging behaviour should not be overlooked, and understanding these would provide insight into disease management approaches.

For both settlements, there was no notable difference in epidemic size between simulations using the observed binomial and weighted networks. This result would suggest that including non-random mixing (whom individuals contact) in disease models is more important than including non-uniform mixing (contact duration/frequency). However, heterogeneities in edge weights are likely to be important and have been shown to further limit epidemic sizes when they are allowed to be dynamic in time [44]. To further understand the effect of non-uniform mixing, future research should try to describe the temporal dynamics of free-ranging dog contacts over a timeframe relevant to the disease in question. Specifically, investigations should look for daily and seasonal differences in network structure and identify whether or not individuals occupy stable network positions.

The model of rabies transmission used in this study makes several assumptions that should be considered. First, individuals do not change their behaviour once infected. It is well known that rabies can manifest as either encephalitic (furious) or paralytic (dumb) and evidence suggests that, unless vaccinated, the furious form is more likely to develop in dogs [45]. However, it is not clear what determines the type of rabies an individual develops or if the different forms result in considerable deviations from the individuals’ typical behaviour. Such deviations could result in changes to the contact network with either new connections being formed, the loss of connections or changes in the strength of connections. A second assumption is that when individuals were removed due to death, the network structure did not change. Removing these assumptions would require a rewiring of the network and this process should be biologically informed. Reynolds et al [7] attempted to account for dumb and furious behaviours by assuming different frequencies of each and either changing the transmission probability (higher for furious and lower for dumb) or by altering the individual’s contact behaviour (removing half their connections for dumb or doubling them for furious). They found that both methods produced similar results and the speed of transmission increased when there was a higher frequency of furious individuals and decreased with a higher frequency of dumb individuals. Although this effort to model behavioural change can be insightful, the methods of rewiring are not biologically informed and so should be interpreted carefully as they cover a limited number of possible scenarios in which the network could change. Solutions to such network dynamics are challenging as there is a lack of experimental data on the processes of network rewiring and, without this guidance, the number of potential modes of change is too computationally demanding to include in models. For diseases such as rabies it is unlikely that such data will ever exist given the ethical implications of such experimentation. However, understanding how a network rewires as individual states or community membership change could better allow network models to include such dynamics that are thought to be a major obstacle for controlling rabies [38].

The inflation of predictions for epidemic size in models that do not account for observed contact heterogeneities are of particular concern when public health resources are limited [46]. This is the case for dog-mediated rabies in developing countries, where epidemics are preventable through vaccination but a major challenge is the high incidence of dog infections and human cases, combined with limited public health resources [18]. Currently it is advised that successful vaccination campaigns require 70% coverage of the dog population [47]. However, through targeted management this might be reduced, helping alleviate costs. Further to work on urban dogs [26], our results show that even in a rural context, epidemic risk is not equal among individuals and suggest that, by identifying the network position of individuals and correlates thereof, targeted management could be feasible. We find evidence to suggest that the spatial ranging behaviour of dogs was associated with their network position, though anthropogenic influences clearly have a role in determining free-ranging dog movements and this deserves further investigation. Our research illustrates how a greater understanding of the social contact network of free-ranging dogs can help better inform the management of diseases such as dog-mediated rabies.

## Supporting information

S1 Table Normalized Mutual Information (NMI) score for the relationship between the community membership of free-ranging dogs in two rural settlements in Chad and their attributes; sex, age and household membership. NMI scores closer to 1 imply a greater overlap between community membership and attributes. Community membership is calculated from observed binomial and weighted contact networks and calculated using both the edge betweenness (EB) and Greedy (G) algorithms.
(DOCX)

S2 Table Duration of epidemics simulated on the contact networks of free-ranging dogs from two rural settlements in Chad. Results are reported for R_0_ set to 1.2, 1.8 and 2.4, and for the random, binomial and weighted networks of the settlements Kakale and Magrao. Summary statistics are for simulations where at least one individual was infected by the seeded individual. The mean ± standard error is reported for the duration of epidemics that ended within the 300 days. The percentage of simulations that were longer than 300 days is also reported.
(DOCX)

S1 Fig Frequency distribution of total ranges recorded for free-ranging dogs from two rural settlements in Chad. Total ranges are 99% Minimum Convex Polygons.
(PNG)

S2 Fig Relationships between the strength of contacts among free-ranging dogs in two rural settlements in Chad and the distance between their respective households. Scatter plots show the logged daily average edge weights between observed dyads in the settlements Kakale and Magrao against the distance (m) between their households. The histograms show the distribution of r coefficients calculated from permutations where edges were randomly shuffled. The red lines on the histograms indicate the observed r coefficient. For all plots, edges for individuals in the same household were excluded.
(PNG)

S3 Fig Number of secondary cases produced from epidemics simulated on the contact networks of free-ranging dogs from two rural settlements in Chad. Density plots for the number of secondary cases in simulations of the different networks (columns) of the settlements Kakale and Magrao and for the different R_0_ values (rows).
(PNG)

S4 Fig Relationship between the proportion of simulations to have an epidemic and the seeded individual’s ranked centrality measures (eigenvector, degree and betweenness) when disease transmission is simulated through the contact networks of free-ranging dogs from two rural settlements in Chad. The scatter plots include the results for the random, binomial and weighted networks of each settlement (Kakale and Magrao), and are for each R_0_ value modelled (1.2, 1.8 and 2.4). GAMs are fitted to the data to identify non-linear trends.
(PNG)

S5 Fig The relationship between the mean epidemic size of simulations and the seeded individuals ranked centrality measures (eigenvector, degree and betweenness) when disease transmission is simulated through the contact networks of free-ranging dogs from two rural settlements in Chad. The scatter plots include the results for the random, binomial and weighted networks of each settlement (Kakale and Magrao), and are for each R_0_ value modelled (1.2, 1.8 and 2.4). The means exclude simulations where the infection did not spread beyond the seeded individual. GAMs are fitted to the data to identify non-linear trends.
(PNG)

S6 Fig The distribution of eigenvector centrality scores for free-ranging dogs from two rural settlements in Chad. Bean plots are plotted for dogs from the settlements Kakale and Magrao.
(PNG)
