# Supplementary material for: High-resolution contact networks of free-ranging domestic dogs Canis familiaris and implications for transmission of infection
Source: PLoS Negl Trop Dis. 2019 Jul 15;13(7):e0007565. doi: 10.1371/journal.pntd.0007565 (PMC6658143; doi:10.1371/journal.pntd.0007565)
Supplement: S1 Table — NMI scores closer to 1 imply a greater overlap between community membership and attributes. Community membership is calculated from observed binomial and weighted contact networks and calculated using both the edge betweenness (EB) and Greedy (G) algorithms. (DOCX) [file pntd.0007565.s001.docx]

**S1 Table. Normalized Mutual Information (NMI) score for the relationship between the community membership of free-ranging dogs in two rural settlements in Chad and their attributes; sex, age and household membership.**

| **Attribute** | **Settlement** | **EB binomial** | **EB weighted** | **G binomial** | **G weighted** |
| --- | --- | --- | --- | --- | --- |
| Sex | *Kakale* | 0.048 | 0.058 | 0.042 | 0.069 |
|  | *Magrao* | 0.077 | 0.062 | 0.053 | 0.066 |
| Age | *Kakale* | 0.196 | 0.147 | 0.070 | 0.147 |
|  | *Magrao* | 0.165 | 0.164 | 0.150 | 0.115 |
| Household | *Kakale* | 0.622 | 0.674 | 0.625 | 0.70 |
|  | *Magrao* | 0.739 | 0.725 | 0.649 | 0.713 |
| NMI scores closer to 1 imply a greater overlap between community membership and attributes. Community membership is calculated from observed binomial and weighted contact networks and calculated using both the edge betweenness (EB) and Greedy (G) algorithms. | | | | | |
