# Supplementary material for: High-resolution contact networks of free-ranging domestic dogs Canis familiaris and implications for transmission of infection
Source: PLoS Negl Trop Dis. 2019 Jul 15;13(7):e0007565. doi: 10.1371/journal.pntd.0007565 (PMC6658143; doi:10.1371/journal.pntd.0007565)
Supplement: S2 Table — Results are reported for R0 set to 1.2, 1.8 and 2.4, and for the random, binomial and weighted networks of the settlements Kakale and Magrao. Summary statistics are for simulations where at least one individual was infected by the seeded individual. The mean ± standard error is reported for the duration of epidemics that ended within the 300 days. The percentage of simulations that were longer than 300 days is also reported. (DOCX) [file pntd.0007565.s002.docx]

**S2 Table: The duration of epidemics simulated on the contact networks of free-ranging dogs from two rural settlements in Chad.**

| Village | R0 | Network | Duration (days) | Duration > 300 (%) |
| --- | --- | --- | --- | --- |
| Kakale | 1.2 | Random | 29 ± 1 | 0.01 |
|  |  | Binomial | 28 ± 1 | 0.01 |
|  |  | Weighted | 32 ± 1 | 0 |
|  | 1.8 | Random | 102 ± 2 | 0.13 |
|  |  | Binomial | 86 ± 1 | 0.05 |
|  |  | Weighted | 95 ± 1 | 0.60 |
|  | 2.4 | Random | 135 ± 2 | 0.09 |
|  |  | Binomial | 112 ± 1 | 0.09 |
|  |  | Weighted | 121 ± 1 | 0.08 |
|  | | | | |
| Magrao | 1.2 | Random | 51 ± 1 | 0.06 |
|  |  | Binomial | 46 ± 1 | 0.02 |
|  |  | Weighted | 52 ± 1 | 0.02 |
|  | 1.8 | Random | 107 ± 2 | 0.20 |
|  |  | Binomial | 84 ± 1 | 0.09 |
|  |  | Weighted | 92 ± 1 | 0.07 |
|  | 2.4 | Random | 139 ± 1 | 0.12 |
|  |  | Binomial | 113 ± 1 | 0.16 |
|  |  | Weighted | 117 ± 1 | 0.14 |
| Results are reported for R_0_ set to 1.2, 1.8 and 2.4, and for the random, binomial and weighted networks of the settlements Kakale and Magrao. Summary statistics are for simulations where at least one individual was infected by the seeded individual. The mean ± standard error are reported for the duration of epidemics that ended within the 300 days. The percentage of simulations that were longer than 300 days is also reported. | | | | |
