# Supplementary figures and images for: High-resolution contact networks of free-ranging domestic dogs Canis familiaris and implications for transmission of infection
Source: PLoS Negl Trop Dis. 2019 Jul 15;13(7):e0007565. doi: 10.1371/journal.pntd.0007565 (PMC6658143; doi:10.1371/journal.pntd.0007565)

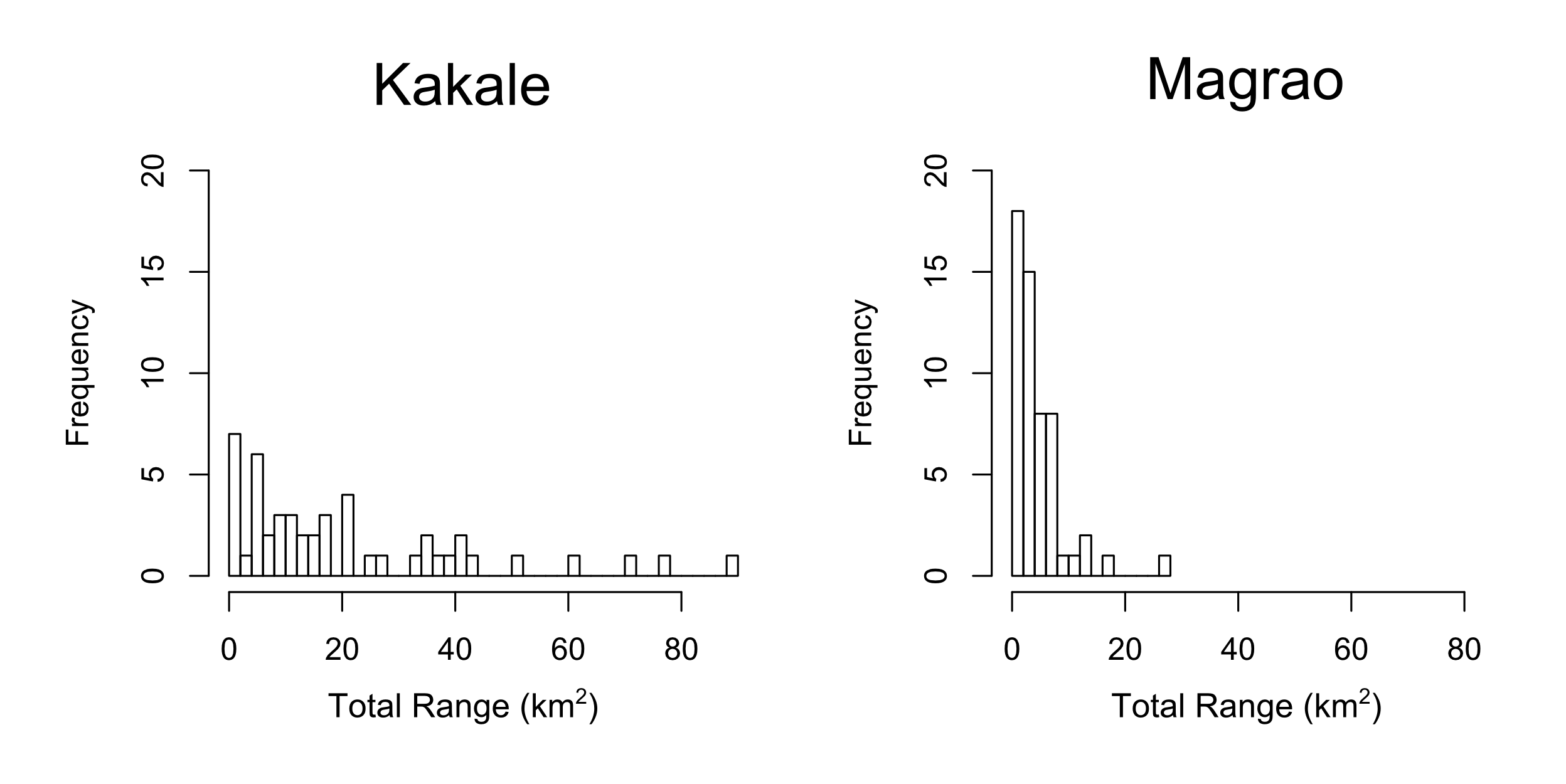

Supplement: S1 Fig — Total ranges are 99% Minimum Convex Polygons. (PNG) [file pntd.0007565.s003.png]

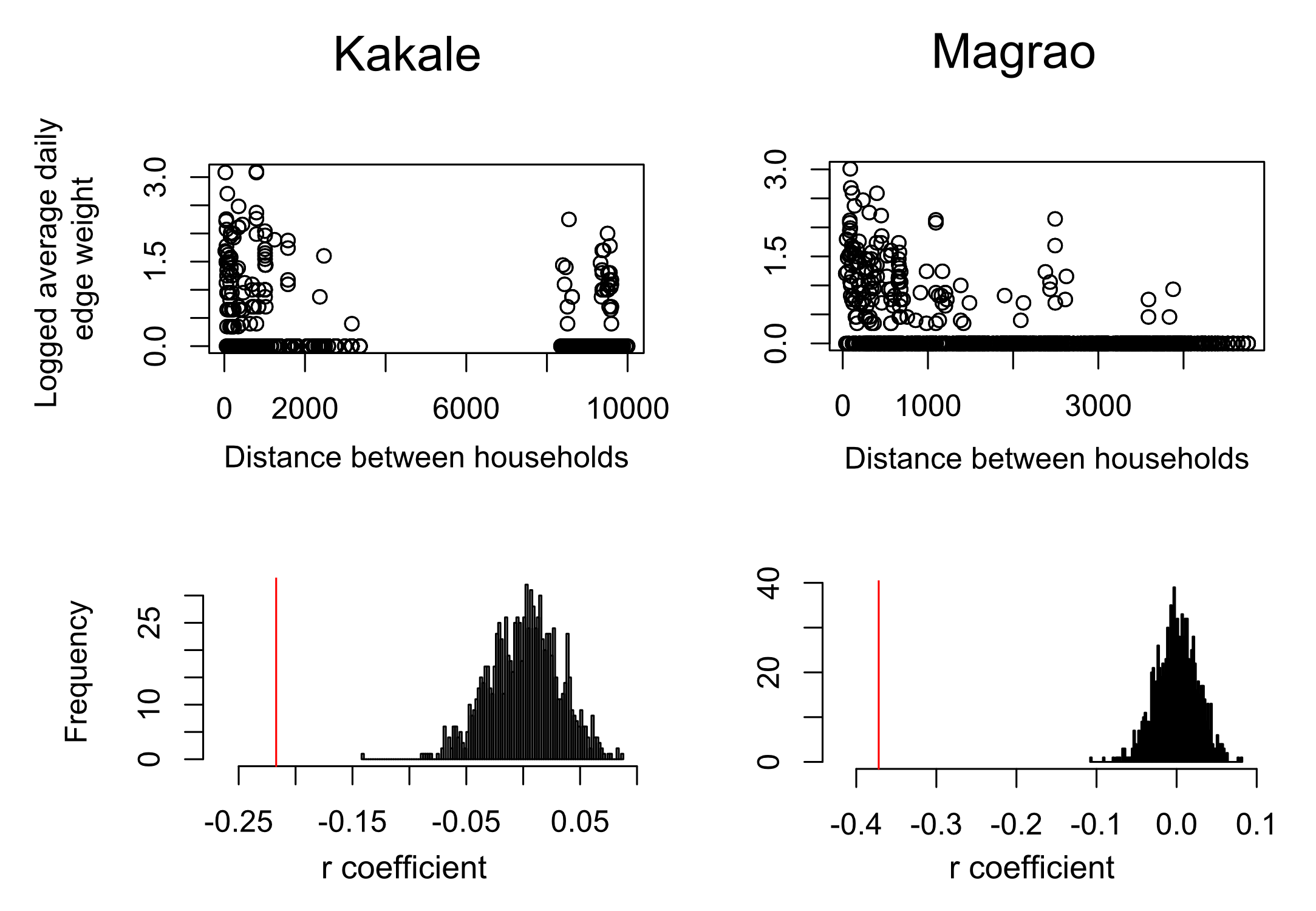

Supplement: S2 Fig — Scatter plots show the logged daily average edge weights between observed dyads in the settlements Kakale and Magrao against the distance (m) between their households. The histograms show the distribution of r coefficients calculated from permutations where edges were randomly shuffled. The red lines on the histograms indicate the observed r coefficient. For all plots, edges for individuals in the same household were excluded. (PNG) [file pntd.0007565.s004.png]

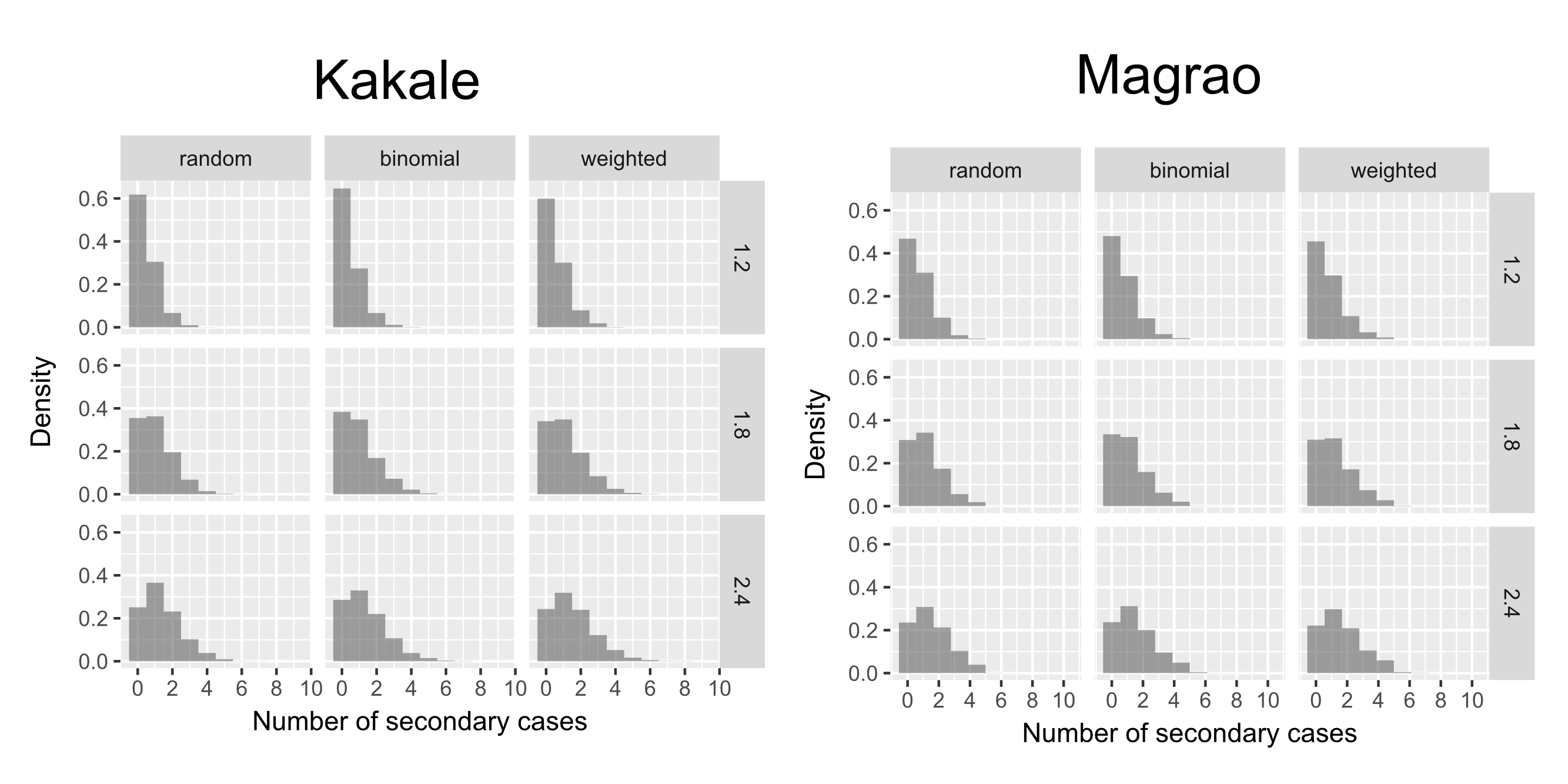

Supplement: S3 Fig — Density plots for the number of secondary cases in simulations of the different networks (columns) of the settlements Kakale and Magrao and for the different R0 values (rows). (PNG) [file pntd.0007565.s005.png]

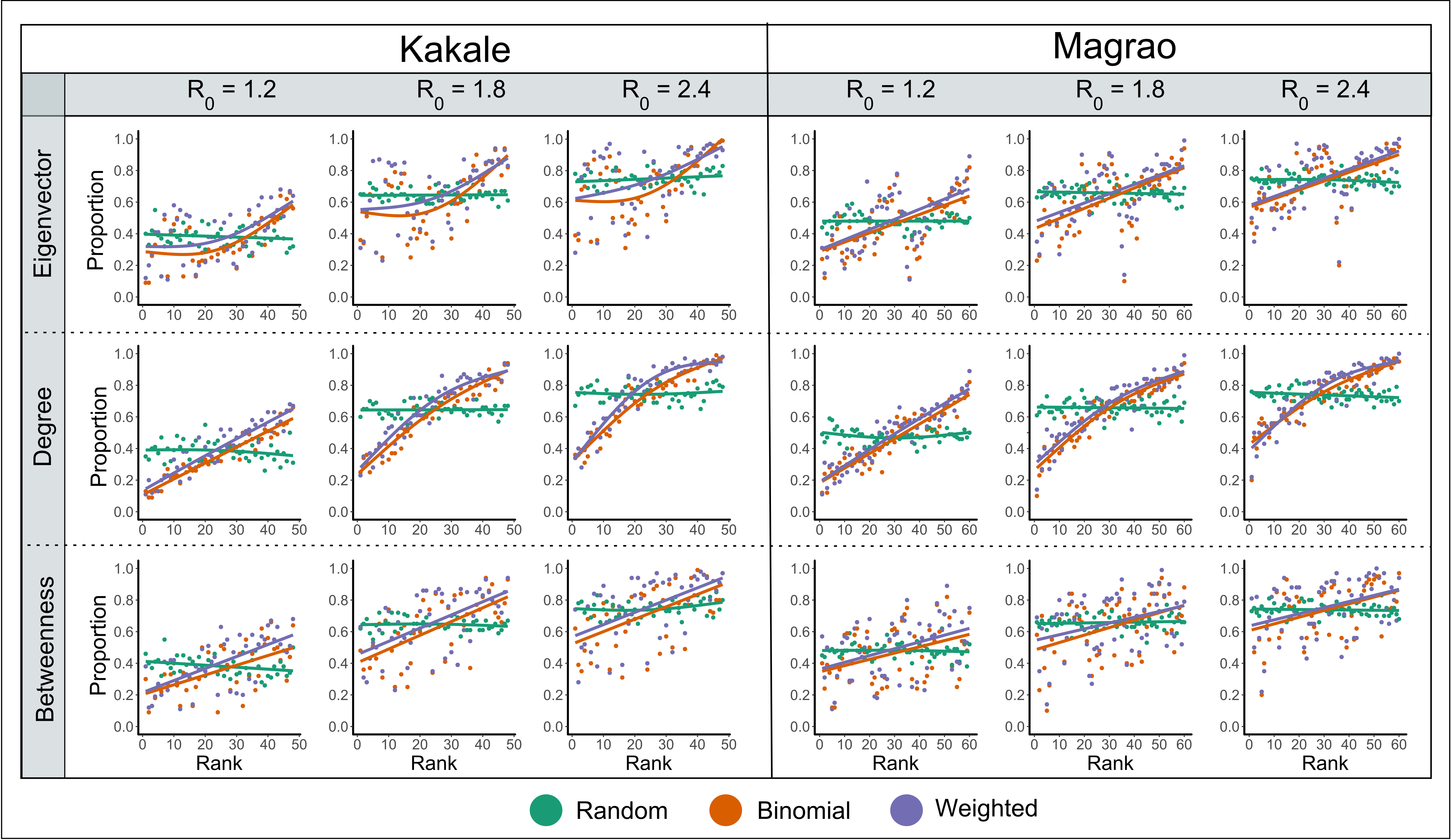

Supplement: S4 Fig — The scatter plots include the results for the random, binomial and weighted networks of each settlement (Kakale and Magrao), and are for each R0 value modelled (1.2, 1.8 and 2.4). GAMs are fitted to the data to identify non-linear trends. (PNG) [file pntd.0007565.s006.png]

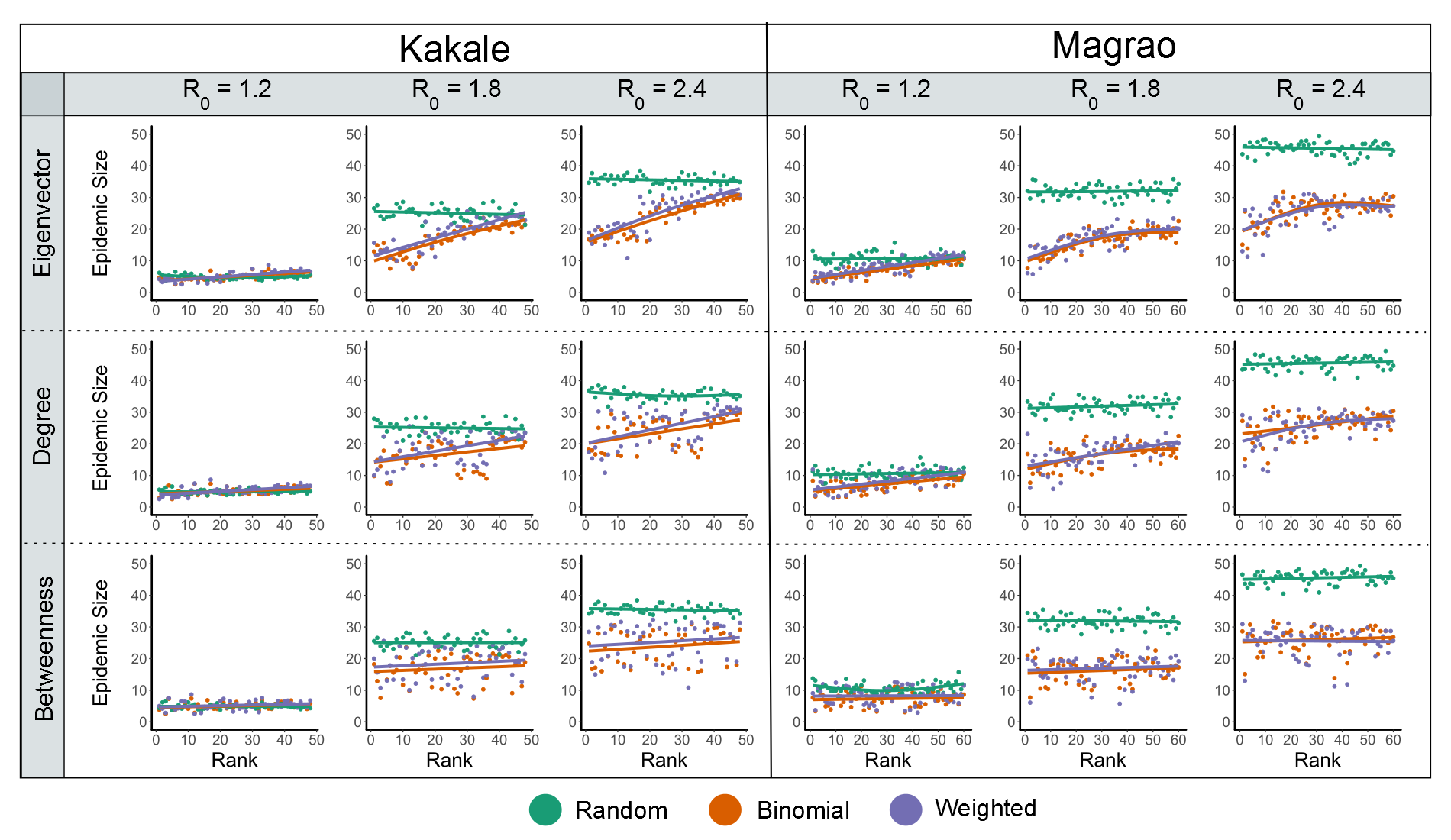

Supplement: S5 Fig — The scatter plots include the results for the random, binomial and weighted networks of each settlement (Kakale and Magrao), and are for each R0 value modelled (1.2, 1.8 and 2.4). The means exclude simulations where the infection did not spread beyond the seeded individual. GAMs are fitted to the data to identify non-linear trends. (PNG) [file pntd.0007565.s007.png]

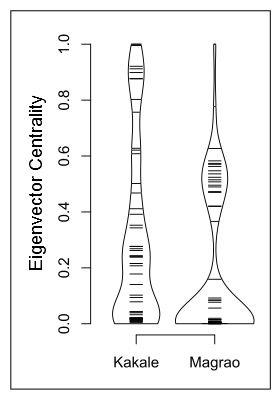

Supplement: S6 Fig — Bean plots are plotted for dogs from the settlements Kakale and Magrao. (PNG) [file pntd.0007565.s008.png]
